# Proactive Management of Intraoperative Hypotension Reduces Biomarkers of Organ Injury and Oxidative Stress during Elective Non-Cardiac Surgery: A Pilot Randomized Controlled Trial

**DOI:** 10.3390/jcm11020392

**Published:** 2022-01-13

**Authors:** Paolo Murabito, Marinella Astuto, Filippo Sanfilippo, Luigi La Via, Francesco Vasile, Francesco Basile, Alessandro Cappellani, Lucia Longhitano, Alfio Distefano, Giovanni Li Volti

**Affiliations:** 1 Department of General Surgery and Surgical-Medical Specialties, Section of Anesthesia, University of Catania, Via S. Sofia 72, 95125 Catania, Italy; marinella.astuto@unict.it (M.A.); filipposanfi@yahoo.it (F.S.); luigilavia7@gmail.com (L.L.V.); frankie.vas@hotmail.it (F.V.); francesco.basile@unict.it (F.B.); alessandro.cappellani@unict.it (A.C.); 2 Department of Biomedical and Biotechnological Sciences, University of Catania, Via S. Sofia 97, 95125 Catania, Italy; lucia.longhitano@unict.it (L.L.); distalfio@gmail.com (A.D.); 3 Center of Excellence for the Acceleration of Harm Reduction—CoEHAR, University of Catania, Via S. Sofia 97, 95131 Catania, Italy

**Keywords:** hypotension, organ injury, surgery, biomarkers, oxidative stress

## Abstract

Background: Intraoperative hypotension is associated with increased postoperative morbidity and mortality. Methods: We randomly assigned patients undergoing major general surgery to early warning system (EWS) and hemodynamic algorithm (intervention group, *n* = 20) or standard care (*n* = 20). The primary outcome was the difference in hypotension (defined as mean arterial pressure < 65 mmHg) and as secondary outcome surrogate markers of organ injury and oxidative stress. Results: The median number of hypotensive episodes was lower in the intervention group (−5.0 (95% CI: −9.0, −0.5); *p* < 0.001), with lower time spent in hypotension (−12.8 min (95% CI: −38.0, −2.3 min); *p* = 0.048), correspondent to −4.8% of total surgery time (95% CI: −12.7, 0.01%; *p* = 0.048).The median time-weighted average of hypotension was 0.12 mmHg (0.35) in the intervention group and 0.37 mmHg (1.11) in the control group, with a median difference of −0.25 mmHg (95% CI: −0.85, −0.01; *p* = 0.025). Neutrophil Gelatinase-Associated Lipocalin (NGAL) correlated with time-weighted average of hypotension (R = 0.32; *p* = 0.038) and S100B with number of hypotensive episodes, absolute time of hypotension, relative time of hypotension and time-weighted average of hypotension (*p* < 0.001 for all). The intervention group showed lower Neuronal Specific Enolase (NSE) and higher reduced glutathione when compared to the control group. Conclusions: The use of an EWS coupled with a hemodynamic algorithm resulted in reduced intraoperative hypotension, reduced NSE and oxidative stress.

## 1. Introduction

Hemodynamic instability represents a relatively common clinical event during surgical procedures. In particular, intraoperative hypotension is a common condition and may cause organ ischemia thus leading to injury and increasing the risk of postoperative complications. In this regard, several studies reported a significant correlation between intraoperative hypotension and organ injury [1,2,3,4]. Although the definition of intraoperative hypotension is variable, it is commonly defined in terms of absolute systolic or mean arterial pressure (MAP) reduction, or relative systolic or MAP reduction from baseline values [5,6]. There is evidence that the risk of harm is due not only to the severity (time and/or duration) of a single event but also to a series of shorter hypotensive episodes; therefore, even relatively short but multiple episodes of hypotension may worsen patient’s outcome. However, the characterization and definition of intraoperative hypotension remains debated and vague. In a systematic review, Bijker et al. [5] found 140 definitions for hypotension reported by 130 articles. Nevertheless, there is a wide agreement regarding the importance of promptly treating intraoperative hypotension with the aim of preventing postoperative complications. Previous studies evaluated if hypotensive events could be predicted allowing clinicians to move from a reactive to a proactive approach, avoiding or reducing the impact of intraoperative hypotension. A recent randomized trial showed that the use of a machine learning–derived early warning system (EWS) as compared with standard care resulted in significantly lower intraoperative hypotension [7]. In this context, the key-player leading to organ injury appears to be the mismatch between oxygen supply and demand [8,9,10]. Furthermore, after periods of ischemia, tissue reperfusion may lead to increased production of oxidative stress (i.e., reactive oxygen species, isoprostanes, lipid hydroperoxides) concurring to organ injury. However, no studies investigated whether such proactive approach is coupled with a reduced organ injury as measured by biomarker assessment. Therefore, we aimed at evaluating the impact of an EWS with an algorithm for hemodynamic management on the intraoperative time spent with hypotension in adult patients undergoing major general surgery; we also assessed the impact of the intervention on post-operative levels of biomarkers of organ injury and oxidative stress.

## 2. Materials and Methods

The present single center pilot randomized clinical trial took place at the “Azienda Ospedaliera Universitaria Policlinico-San Marco, site Gaspare Rodolico”, a tertiary academic center. The study was approved by the local institutional review board (Ethics committee “Catania 1”—reference protocol number: NL62115.018.17). The study was registered on www.clinicaltrialgov (NCT03527758) on 17 May 2018.

### 2.1. Patients

We randomized adult patients (≥18 years old) undergoing elective laparotomic major general surgery under general anesthesia if an intraoperative continuous invasive blood pressure monitoring was planned. Written informed consent was obtained from all patients before surgery. The first participant was enrolled in January 2019, and the last follow-up was in January 2020. A phone interview was performed 30 days following surgery in order to record the onset of late postsurgical complications and/or hospital readmissions. A target MAP of at least 65 mmHg during surgery was mandatory. Patients for whom the attending anesthesiologists requested a different MAP target were excluded. Other exclusion criteria were: emergency surgery, patients with cardiac failure or cardiac shunts, severe aortic stenosis, or preoperative cardiac arrhythmias (in accordance with the summary of product characteristics of the EWS), hypotension (MAP < 65 mmHg) before surgery, acute or chronic renal disease, need for vascular clamping during surgical procedure.

### 2.2. Randomization

**Figure 1:**
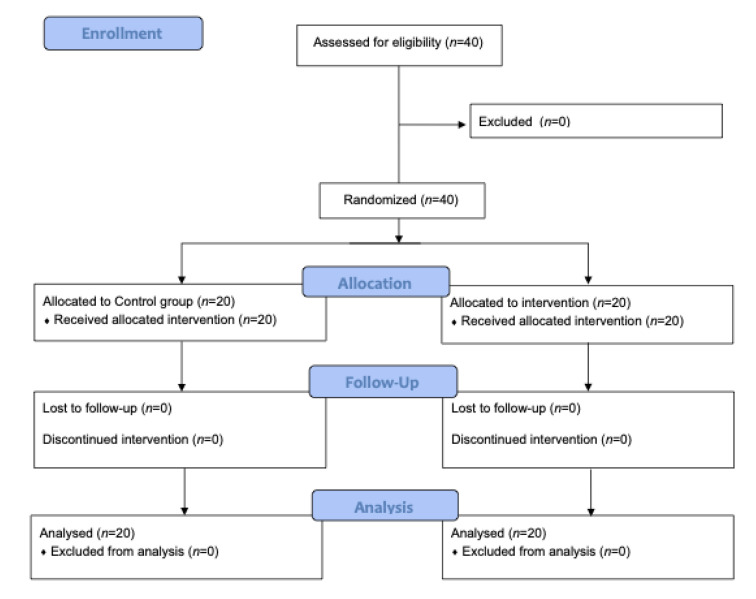
Consort flow diagram describing different phases of the present pilot trial.

Patients were randomized into two different groups (*n* = 20 each): the intraoperative EWS monitoring coupled with an algorithm for hemodynamic management (intervention group) or standard care (control group) (Figure 1). A computer-generated permutated block randomization (concealed and varying permutated block sizes of 4 and 6 patients) was used with a 1:1 allocation ratio. The researchers involved in the study for laboratory measurements and statistical analyses were blinded to group allocation.

### 2.3. Procedure

A radial artery catheter was placed in all patients prior to the induction of general anesthesia and it was connected to the FloTrac IQ sensor with EWS software in the intervention group, and to standard FloTrac sensor (both marketed by Edwards Lifesciences^®^) in the control group. System zeroing and quality control of the arterial signal was performed by the anesthesiologist. The arterial pressure waveform was measured continuously with a sample frequency of 100 Hz. The standard FloTrac and the FloTrac IQ pressure transducers were connected to the HemoSphere monitor (Edwards Lifesciences^®^ hereafter referred to as the advanced hemodynamic monitor), and the resulting electrical signal was transmitted to a Mindray^®^ monitor (hereafter referred to as the standard monitor). The HemoSphere monitor displayed commonly used hemodynamic parameters (Stroke Volume, Stroke Volume Variation, Cardiac Index, Systemic Vascular Resistance) calculated from the waveform every 20 s, as well as the Hypotension Prediction Index (HPI) (updated every 20 s). The standard monitor displayed the MAP, systolic arterial pressure, and diastolic arterial pressure. In the control group, the advanced hemodynamic monitor was connected in order to collect the intraoperative data, but the screen was fully covered, and the alarms were silenced; anesthesiologists solely used the variables visible on the standard monitor (i.e., invasive blood pressure) to guide hemodynamic treatment.

**Figure 2:**
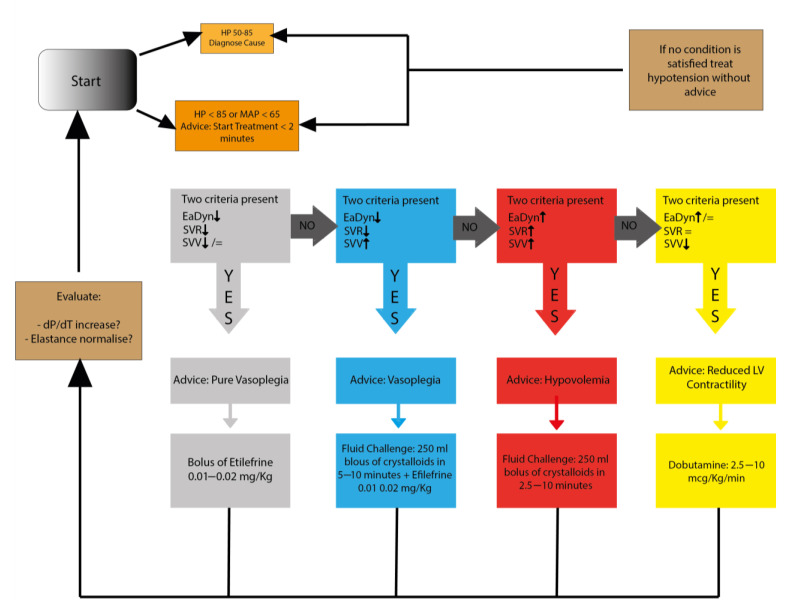
Algorithm for hemodynamic management used in the intervention group and coupled with the hypotension prediction index. Arrows indicate the clinical decision to be followed according to patient parameters.

Upon reaching the predefined MAP threshold (<65 mmHg), the anesthesiologist treated the patient in accordance with a standardized algorithm in use at our Hospital. In the intervention group, when the HPI value was greater than 85%, the anesthesiologists applied a proactive algorithm for hemodynamic management after a check for artifacts; (Figure 2) the algorithm was based on the values of advanced hemodynamic parameters (Stroke Volume Variation, Dynamic Arterial Elastance, Maximum Delta Pressure over Delta Time). In both groups, each pharmacological intervention was recorded by an external observer. At the end of the procedure, all the data were downloaded from the advanced hemodynamic monitor.

### 2.4. Outcomes

Our primary outcome was the evaluation of hypotension (defined as MAP < 65 mmHg) incidence in the two groups. Secondary outcomes were:

- Number of hypotensive events;
- Absolute time spent with hypotension during surgery (minutes);
- Time spent in hypotension relative to surgical duration (%);
- Time-weighted average of hypotension during surgery; measured by calculating the area under the threshold divided by the total duration of surgery. Practically, this parameter is calculated as the maximum depth of hypotension below the threshold of MAP < 65 mmHg (unit: mmHg) × total time spent in hypotension (unit: minutes) divided by total duration of surgery (unit: minutes). For example, a patient undergoing surgery lasting 100 min experiences 5 episodes of hypotension, all of them lasting 1 min, and all with a minimal MAP of 60 mmHg. In this case, the area under the threshold is 25 mmHg per minute (calculated as 5 min of hypotension ×  5 mmHg of MAP < 65 mmHg). Finally, the time-weighted average will be 25 mmHg per minute divided by 100 min of surgery, corresponding to 0.25 mmHg.

The secondary outcome was also to compare the levels of biomarkers of brain, myocardial and kidney injury and oxidative stress in both groups. Blood samples were collected at three time-points from the radial artery: prior to the induction of general anesthesia (T0), 2 h after (T1) and at the end of the surgical procedure (T2). Samples were allowed to clot for 30 min at room temperature before centrifugation for 15 min at 1000× *g*. Serum was then immediately separated in aliquots and stored at −80 °C to avoid repeated freeze-thaw cycles. A blood gas analysis was immediately performed after blood sample collection. For the purposes of this first preliminary study, only the samples obtained at T0 and T2 were used.

Biomarkers of major organs including the brain (neuron-specific enolase (NSE) and S100B protein), heart (high-sensitive troponin (hsTPN), and kidney (neutrophil gelatinase-associated lipocalin (NGAL) were assessed in all patients. Hypoxia-inducible factor-1 alpha (HIF-1α), acetyl-CoA reduced glutathione (GSH), lipid hydroperoxide (LOOH) were used to determine hypoxia-mediated pathway and oxidative stress status of patients. Biomarkers of organ injury were chosen on the basis of their validation in a clinical setting and their specificity of organ injury. On the other hand, oxidative stress markers were selected on the basis on our previous experience with methodology in our laboratories and sample handling.

### 2.5. Statistical Analysis

Based on previously published studies [1,3,10] and an expected reduction of incidence of hypotension from 80% to 38%, it was calculated that a sample size of 40 patients, 20 in each group, would have 80% power to detect this effect using a 2-group *t* test with an α = 0.05, at 2-sided significance level.

SPSS version 17^®^ was used to perform the statistical analysis. Categorical data are presented as frequencies and percentages. Differences between categorical data were analyzed using the Chi-Square test. Distribution of values was tested for normality with Kolmogorov-Smirnov test. Continuous data are presented as medians with interquartile range (IQR). The difference of hypotension-related outcomes, as well as the pre- post-surgery variation of biomarkers of inflammation and hypoxia between intervention and control group are presented as median differences and 95% confidence intervals (CIs) calculated with the Hodges-Lehmann method. The differences between continuous data were analyzed using the Mann-Whitney U test. The Spearman’s correlation coefficients were calculated to investigate the relation between biochemical markers of inflammation and hypotension-related outcomes. For each of the analyses, a 2-tail probability value of *p* < 0.05 was considered to be statistically significant.

## 3. Results

### 3.1. Study Population

As shown in Table 1, all the baseline characteristics did not differ between groups. In particular, the majority of patients underwent gastrointestinal surgery and no differences in surgery duration was observed between groups.

### 3.2. Hypotension-Related Outcomes

The intervention group had a lower number of hypotensive events than the control group (3 (IQR 6) vs. 8 (IQR 8) median times over surgery time respectively), with lower time of surgery spent in hypotension (both absolute time and time relative to surgical duration, Table 2). The median difference of the incidence of hypotensive episodes was −5.0 episodes (95% CI −9.0, −0.5; *p* < 0.001), with a median difference of total time spent in hypotension of −12.8 min (95% CI −38.0, −2.3 min; *p* < 0.001), correspondent to −4.8% of total surgery time (95% CI −12.7, 0.01%; *p* = 0.048) (Table 2).

The median time-weighted average of hypotension was 0.12 mmHg (IQR, 0.35 mmHg) in the intervention group and 0.37 mmHg (1.11 mmHg) in the control group, with a median difference of −0.25 mmHg (95% CI: −0.85, −0.01; *p* = 0.025; Table 2). All patients survived and following our telephone interview, only one patient in the control group referred hospital readmission because of atrial fibrillation. No other complications were referred for the intervention group.

### 3.3. Biochemical Markers of Organ Injury and Oxidative Stress

Biochemical markers of organ injury and oxidative stress confirmed to a various extent a correlation with hypotension-related outcomes (Table 3).

Specifically, NGAL correlated with time-weighted average of hypotension (R = 0.316; *p* = 0.038), and S100B protein correlated with all the primary outcomes investi-gated (episodes of hypotension, R = 0.584; *p* < 0.001; absolute time spent in hypoten-sion, R = 0.628; *p* < 0.001; time spent in hypotension relative to surgical duration, R = 0.612; *p* < 0.001; time-weighted average of hypotension, R = 0.575; *p* < 0.001).

The median difference of biochemical markers of oxidative stress between intervention and control group revealed that the intervention group had lower NSE (difference: −0.815, 95% CI: −1.485, −0.229; *p* = 0.045) and higher GSH values (2.6, 95% CI: 0.9, 4.6; *p* = 0.033). The difference in S100B protein (−0.8, 95% CI: −1.2, −0.4, *p* = 0.62) did not reach statistical significance (Table 4).

## 4. Discussion

Our pilot study investigated the ability of a machine learning–derived EWS in combination with an algorithm for hemodynamic management in reducing hypotension episodes and duration during major general surgery. Furthermore, we evaluated the clinical impact of intraoperative hypotension on end-organ damage biomarkers and oxidative stress with a series of biochemical assays.

We found that the intervention group exhibited significantly reduced incidence and duration of intraoperative hypotension, as well as lower time-weighted average of hypotension during surgery. Our results are consistent with previous studies showing that the EWS was able to predict hypotension with good sensitivity and specificity [10,11]. To the best of our knowledge, our study is the first one demonstrating that the use of an EWS with a hemodynamic algorithm with the consequent reduction in intraoperative hypotension was associated also with a significant reduction in the value of biomarkers of organ injury and oxidative stress.

Our results add knowledge to the growing body of evidence regarding the role of HPI in predicting intraoperative episodes of hypotension, thus allowing a proactive anesthesiologic approach. Once an alarm was detected (HPI > 85%), the anesthesiologist in charge followed a treatment algorithm based on advanced hemodynamic parameters, which suggested vasopressor, fluid and/or inotrope administration (alone or in combination), or eventually observation. We found a significant reduction in hypotension in the intervention group, and this was consistent with all the measures performed (episodes, absolute and relative time of hypotension, and time-weighted average). The time-weighted average of hypotension seems a very promising variable to evaluate the degree of intraoperative hypotension, considering both the severity of hypotension and its duration as well as the total surgical time. In this regard, our intervention group found similar values of time-weighted average (0.12 mmHg) as compared to other larger studies (0.10 mmHg [10] and 0.14 mmHg [12]). Indeed, our results seem in line with most of currently published findings. Although a recent study by Maheshwari et al. conducted in 214 patients undergoing non-cardiac surgery questioned the role of HPI [12], most of the published evidence supports the value of HPI in predicting hypotension. Indeed, such results have been found in patients undergoing total hip arthroplasty [13], major/general surgery [14,15] and cardiac surgery [16]. Such studies have demonstrated good sensibility and specificity in predicting hypotension from 5 min (both around 85%) to 15 min (both around 75–80%) before the hypotensive episode [14,16,17]. Of note, two of these studies evaluated the use of HPI throughout non-invasive arterial pressure waveform, with encouraging findings [15,17].

The originality of our study relies on the evaluation of the biochemical aspects of hypotensive episodes, thus functioning as a pilot study for the investigation of the impact of intraoperative hypotension on oxidative stress and end-organ injury. However, the reliability of our findings is limited by the small sample size and the correlation between the primary outcome measures and several biomarkers may warrant further investigation.

Firstly, we showed that HIF-1α is not significantly up-regulated in both groups and is not dependent on the number and duration of hypotensive events. Subunit α is oxygen-sensitive, during normoxia is associated with von Hippel-Lindau (VHL) protein, which is responsible for the induction of its proteasome degradation [18,19]. Therefore, in normoxia, the half lifetime of HIF-1α is greatly shorted than in hypoxia conditions [20], as low pressure of oxygen is responsible for blocking the binding of VHL and HIF-1α and its degradation is then inhibited [21]. However, it should be noted that HIF-1α is generally up-regulated during chronic hypoxic conditions [22] and therefore it is conceivable that hypotension, despite causing a possible oxygen supply–demand mismatch, is not sufficient in terms of duration and oxygen delivery to trigger the HIF-1α pathway. This hypothesis is consistent with our data showing non-significant changes in lactate and acetyl-CoA levels in both groups of patients, thus suggesting that cells are not metabolically rewiring toward a hypoxic phenotype. On the other hand, we observed a significant reduction of the reduced form of GSH in the control group when compared to the intervention group, thus suggesting that hypotensive episodes increased oxidative stress and that the application of a machine learning–derived EWS for pending intraoperative hypotension in combination with a hemodynamic diagnostic guidance may be sufficient in preventing such condition. These results are also consistent with previous reports showing that oxidative stress is a variable and common condition occurring during surgical procedures [22]; however, other variables (i.e., ischemia/reperfusion, surgical procedures, anesthesia protocols) rather than hypotension have been advocate in order to explain intraoperative oxidative stress. Interestingly, non-significant changes were observed for lipid peroxidation markers. Two non-mutually exclusive hypotheses may be responsible to explain such result. The first is that the reduced GSH and its enzymatic biosynthetic machinery is sufficient to counteract the production of lipid hydroperoxides. To this regard, we should note that we excluded from the study those patients affected by chronic diseases which are also responsible for imbalanced glutathione synthesis (i.e., liver diseases). The second possibility is that other more instable adducts are produced downstream the lipid peroxidation pathway (i.e., hydroxynonenal, malonyldhyaldheyde, etc.) and are therefore not detected by the used analysis in the present study. Furthermore, NGAL and troponin were not significant in the intervention group when compared to controls. Interestingly, we also observed a significant reduction of NSE in the intervention group when compared to control. Furthermore, increased circulating NSE levels seems to be specific of intraoperative hypotensive events since previous reports showed that NSE was not significantly changed following hypotension in cardiac arrest patients [23]. However, it should be noted that hypotensive events during intraoperative procedures are clinically different from those occurring during cardiac arrest in terms of number of episodes and duration. On the other hand, increased NSE levels, but not S100B, were observed in patients undergoing controlled hypotension during skull base procedures [24]. These results are also consistent with our observations regarding S100B levels. To this regard, even though S100B did not reach statistical significance it showed a trend similar to NSE. It is possible that an increased sample size would clarify this issue. Finally, previous reports showed that both biomarkers of brain injury can cross blood-brain barrier with different intensity, irrespective of differences in their molecular weight (NSE > S100B) [25]. However, it remains to be determined whether increased NSE levels are dependent on increased troponin levels following cardiac impairment or it is a direct consequence of increased oxidative stress mediators formed outside the central nervous system [26,27,28]. Studies using near infrared spectroscopy associated with intraoperative events monitoring are currently running in our unit to further elucidate this point. Finally, our study did not include a long-term neurological examination of enrolled patients and therefore it is not possible to determine whether increased NSE levels are also associated to the clinical outcome in the postoperative period.

Taken all together, our study suggests the importance of monitoring and preventing intraoperative hypotension, reinforcing the clinically meaningful impact that its occurrence has on end-organ damage and oxidative stress. Future multicenter studies on a larger cohort of patients are now warranted to fully elucidate the clinical effectiveness of hypotension prevention on organ injury.

### Limitations

Our study has several limitations that should be considered. First, it is a single center study in a relatively small sample size and results should be interpreted with caution. Larger and multicenter studies are warranted to support or not our findings. Second, the study was not powered enough to investigate the correlation of intraoperative hypotension with short and long-term postoperative complications. Third, it remains to be studied if the higher values of several biomarkers of organ injury and oxidative stress truly correlates with postoperative organ damage and with patient’s outcome. Forth, we included a heterogeneous sample of patients undergoing major non-cardiac surgery and with different perioperative risk stratification. Considering the volume of surgery at our Institution it would have not been feasible to enroll patients undergoing the same surgical operation.

## 5. Conclusions

The use of an EWS monitoring on the risk of hypotension coupled with a hemodynamic algorithm significantly reduced the occurrence and duration of intraoperative hypotension. Patients randomized to EWS monitoring had lower postoperative values of NSE and oxidative stress.

## Figures and Tables

**Table 1:** Background characteristics of the intervention and control groups. ASA: American Society of Anesthesiologist. BMI: Body Mass Index. * Included nephrectomy and removal of abdominal liposarcoma.

	Intervention	Controls	*p*-Value
Age (years, median)	69.0	70.5	0.39
BMI, (Kg/m^2^, median)	25.3	25.6	0.15
Gender, *n* (%)			
Men	10 (50%)	12 (60%)	0.62
Women	10 (50%)	8 (40%)	
ASA classification, *n* (%)			
I	1 (5%)	1 (5%)	0.94
II	8 (40%)	9 (45%)	
III	11 (55%)	10 (50%)	
Type of surgery, *n* (%)			
Gastrointestinal	18 (90%)	16 (80%)	0.81
Gynecological	1 (5%)	2 (10%)	
Others *	1 (5%)	2 (10%)	
Comorbidities, *n* (%)			
Hypertension	10 (50%)	14 (67%)	0.22
Type-2 diabetes	2 (10%)	4 (19%)	0.35
Others	8 (40%)	7 (33%)	0.49
Surgery duration, min (median)	207.0 (64.0)	237.0 (121.0)	0.18

**Table 2:** Comparison of hypotension-related outcomes in the intervention and control group.

	Median (IQR)		Median Difference	95% CI		*p*-Value
	Intervention	Controls		Lower	Higher	
Number of hypotensive episodes, *n*	3 (6)	8 (13)	−5.0	−9.0	−0.5	<0.001
Total time spent in hypotension, min	4.3 (11)	21.3 (28)	−12.8	−38.0	−2.3	<0.001
Time in hypotension relative to surgical duration, %	3.1 (6.4)	7.8 (13.7)	−4.8	−12.7	0.01	0.048
Time-weighted average of hypotension, mmHg	0.12 (0.35)	0.37 (1.11)	−0.26	−0.85	−0.01	0.025

CI: confidence interval; IQR: interquartile range.

**Table 3:** Correlation between biochemical markers of inflammation and hypotension-related outcomes.

	NGAL	NSE	HIF-1α	S100B	Acetyl-CoA	hs Cardiac Troponin	LOOH	GSH	Hypotensive Episodies
NGAL	1.000	-	-	-	-	-	-	-	-
NSE	0.238	1.000	-	-	-	-	-	-	-
HIF-1α	−0.284	−0.369 *	1.000	-	-	-	-	-	-
S100B	0.036	0.258	−0.022	1.000	-	-	-	-	-
Acetyl-CoA	−0.540 **	−0.241	−0.426 **	0.123	1.000	-	-	-	-
Hs Cardiac Troponin	0.356 *	0.337 *	−0.304	0.030	−0.391 *	1.000	-	-	-
LOOH	−0.136	−0.103	−0.40	−0.258	−0.010	−0.040	1.000	-	-
GSH	0.041	−0.213	0.253	−0.283	0.114	−0.398 **	−0.099	1.000	-
Hypotensive Episodies	0.181	0.207	−0.104	0.584 **	−0.027	0.088	−0.133	−0.132	1.000
Absolute time of hypotension	0.288	0.159	−0.133	−0.628 **	−0.111	−0.138	−0.121	−0.164	0.883 **
Relative time of hypotension	0.278	0.118	−0.148	0.612 **	−0.096	0.127	−0.199	−0.179	0.880 **
Time-weighted average of hypotension	0.316 *	0.093	−0.150	0.575 **	−0.153	0.174	−0.160	−0.170	0.820 **

* *p* < 0.05 (2-tailed) Mann-Whitney U test; ** *p* < 0.001 (2 tailed), Mann-Whitney U test. HIF: hypoxia inducible factor; GSH: reduced glutathione; LOOH: lipid hydroperoxide; NGAL: neuthrophil gelatinase-associated lipocalin; NSE: neuron-specific enolase.

**Table 4:** Differences between pre- and post- surgery of biochemical markers of inflammation in intervention and control groups.

	Intervention		Controls					
	Pre Median (IQR)	Post Median (IQR)	Pre Median (IQR)	Post Median (IQR)	Median Difference	Lower 95% CI	Upper 95% CI	*p*-Value
NGAL (ng/mL)	2.1 (1.7)	2.2 (1.8)	2.2 (1.4)	2.0 (1.0)	0.24	−0.27	0.84	0.528
NSE (ng/mL)	2.0 (1.3)	1.0 (1.0)	1.9 (1.9)	1.7 (1.8)	−0.81	−1.48	−0.23	0.045
HIF-1alpha (ng/mL)	0.18 (0.12)	0.15 (0.05)	0.16 (0.11)	0.15 (0.04)	−0.003	−0.02	0.02	0.766
S100B (pg/mL)	0.36 (0.34)	0.51 (0.63)	0.32 (2.0)	1.36 (1.29)	−0.76	−1.21	−0.42	0.622
Acetyl–CoA (pmol/μL)	0.11 (0.23)	0.30 (1.4)	0.16 (0.48)	0.35 (2.0)	−0.09	−0.84	0.36	0.509
Human Cardiac Troponin 1 (pg/mL)	41.8 (32.7)	34.9 (36.9)	32.1 (36.3)	39.9 (39.2)	−4.58	−31.2	4.7	0.584
LOOH (nmol/μL)	6.1 (7.6)	4.4 (7.1)	4.4 (5.0)	3.9 (4.8)	−0.12	−3.1	2.9	0.969
GSH (nmol/μL)	7.5 (7.0)	7.0 (3.7)	7.0 (5.5)	5.0 (2.5)	2.62	0.89	4.61	0.033

HIF: hypoxia inducible factor; IQR: interquartile range; GSH: reduced glutathione; LOOH: lipid hydroperoxide; NGAL: neuthrophil gelatinase-associated lipocalin; NSE: neuron-specific enolase. Differences were evaluated with Mann-Whitney U test.

## Data Availability

Not applicable.
